# Age, puberty, body dissatisfaction, and physical activity decline in adolescents. Results of the German Health Interview and Examination Survey (KiGGS)

**DOI:** 10.1186/1479-5868-8-119

**Published:** 2011-10-27

**Authors:** Emily Finne, Jens Bucksch, Thomas Lampert, Petra Kolip

**Affiliations:** 1 Bielefeld University, School of Public Health, PO Box 10 01 31, D-33501 Bielefeld, Germany; 2 Robert Koch Institute, Department of Epidemiology and Health Reporting, General-Pape-Str. 62-66, D-12101 Berlin, Germany; 3 University of Stuttgart, Department of Sport and Exercise Science, Allmandring 28, D-70569 Stuttgart, Germany

**Keywords:** physical activity, adolescents, sexual maturation, pubertal timing, body dissatisfaction

## Abstract

**Background:**

Physical activity (PA) shows a marked decline during adolescence. Some studies have pointed to pubertal status or timing as possible PA determinants in this age group. Furthermore, it was supposed that the impact of pubertal changes on PA might be mediated by psychological variables like body dissatisfaction (BDS).

**Methods:**

The 11- to 17-year-old subsample of the German Health Interview and Examination Survey (KiGGS) was used (n = 6 813; 51.3% male, response rate = 66.6%). Through sex-specific sequential multinomial logistic regressions we analysed the univariate and independent associations of chronological age, absolute pubertal status, relative pubertal timing, and BDS with the frequency of PA.

**Results:**

Chronological age showed a significantly negative association with PA in both sexes, independent of puberty. The odds of inactivity in contrast to nearly daily PA increased about 70% in boys and 35% in girls for each year of age, respectively. Adjusted for age and other possible confounders, inactivity was significantly less likely for boys in late pubertal stages (OR = 0.27, 95% CI = 0.09-0.78). The risk of inactivity was more than doubled in boys maturing earlier than peers in terms of relative pubertal timing (OR = 2.20, 95% CI = 1.36-3.56). No clear significant puberty effects were found in girls, but the inactivity was more likely for those with irregular menstruation (OR = 1.71, 95% CI = 1.06-2.75). BDS also contributed to the prediction of PA in both sexes. It partially mediated puberty effects in boys but not in girls.

**Conclusions:**

Overall, chronological age was a far more important predictor of PA in German adolescents than absolute pubertal status or relative pubertal timing. Further possible explanatory variables like sociocultural influences, social support or increasing time requirements for education should be analysed in conjunction with chronological age in future studies.

## Background

Various health benefits of physical activity (PA) have been demonstrated in adolescents [1]. Despite this, most youths from Europe are not sufficiently active to meet current PA recommendations [2]. Furthermore, the adolescent years have been identified as a risk period for declining PA levels in studies using different methods, including accelerometry [3]. In Germany, only PA prevalence rates based on self-report data have been published so far, but these point in the same direction with boys being physically more active than girls and a steep decline occurring in PA during adolescence for both. The 2005/2006 WHO-Health Behaviour in School-aged Children study showed that the proportion of German adolescents fulfilling the PA guidelines of 60 minutes of moderate-to-vigorous PA on most days of the week fell from 25% in 11-year-old boys and 20% in girls to only 16% in 15-year-old boys and 10% in girls, respectively [4]. In addition, the percentage of girls and boys who reported daily exercise in the German Health Interview and Examination Survey for children and adolescents (KiGGS) fell from 27.6% in girls and 34.9% in boys at the age of 11 to 11.2% and 18.4% at the age of 17, respectively [5].

Understanding factors responsible for the PA decline in adolescence is crucial for the promotion of PA. Most studies have only described the PA decline during adolescence in terms of chronological age whilst neglecting pubertal maturation. However, puberty is a major life event accompanied by simultaneous rapid changes with respect to biology, physical appearance, social and psychological capabilities [6]. These changes are often associated with health risk behaviours, psychosomatic syndromes and may also be highly relevant to PA behaviour [7-9]. However, compared to other health behaviours only a small number of studies have been conducted on this issue.

In girls a negative influence of the degree of pubertal maturation on PA is to be expected. First of all, girls' increase in body fat is not matched by an increase in lean body mass, whereas in boys there is an increase in muscle mass during puberty with the result of a performance advantage in different kinds of sports. Second, the storage of fat in certain body parts of girls (e.g. hips, thighs, buttocks) results in a deviation from the current western female beauty ideal [10,11]. Boys, in general, tend to approximate an athletic male ideal, but the social pressure to reach this ideal seems to be increasing, too. Bodily changes may impact psychological outcomes in terms of body dissatisfaction (BDS) in adolescents of both sexes [12-14]. Concurrently, physical self-perceptions are one of the key correlates of PA, especially for girls [15]. Several hypotheses and studies also point to the importance of the timing of puberty (relative to same-aged peers), instead of absolute state of pubertal maturation for the choice of health behaviours and for body image [e.g. [9,16]] with partially different implications for boys and girls [9,10,17].

Studies which examined the association between puberty and PA revealed inconsistencies. Some studies found PA levels varying with absolute pubertal status [7,18,19] or with pubertal timing in relation to same-aged peers [16,20], sometimes with different effects in both sexes. Other studies could not find any association between puberty and PA [21-24].

In their longitudinal study Davison and colleagues [25] found that a PA decline in early maturing girls was partially mediated by weight- and shape-related maturity fears, low self-worth, and depression. Another study highlighted an association between the deterioration of physical self-perceptions and the decrease of PA over 12 months in early adolescent girls, but maturation only had a limited influence on PA behaviour [22]. These results point to the importance of psychological factors, like body image, as possible reactions to physical maturation that may foster the PA decline.

The aim of the present study was to shed further light on the complex relationships between chronological age, pubertal maturation, BDS, and PA using data of a large-scale representative sample of German youths. According to the comments above our objectives were to examine

a) the associations between chronological age, absolute pubertal status, relative pubertal timing, and PA

b) the role of BDS as a possible mediator.

We distinguish two concepts of pubertal development. With absolute pubertal status we refer to the stage of secondary sex characteristic development of boys or girls at a given time regardless of what is the average progress of sexual development at this age. In addition, we introduce pubertal timing as the extent of sexual maturation that a boy or girl has reached in relation to others of the same year of age. The latter refers to adolescents being relatively early, late or average ('on-time') maturing when compared to others of the same sex and age within our sample. Pubertal status does not imply this comparison, but refers to the absolute state of development (i.e. degree of pubertal hair growth or voice change independent of the average degree).

Due to sex-specific physiological changes occurring during puberty (i.e. increase in muscle mass in boys versus increase in fat mass in girls) we expected a negative association of pubertal development with PA in girls, while in boys a positive association was expected after controlling for chronological age. In terms of pubertal timing we expected negative effects in 'off-time' youths, especially in early maturing girls and late maturing boys, related to PA and BDS. We also expected lower PA levels for youths dissatisfied with their weight.

## Method

### Sample and procedures

Data from the German Health Interview and Examination Survey (KiGGS) were analysed.

On the whole, 17 641 boys and girls aged 0-17 years and their parents participated in the survey from May 2003 to May 2006. Aims and methodology of the survey are described in detail elsewhere [26].

In brief, a stratified multistage probability sample representative for this age group in Germany was obtained. In 167 local study centres, boys and girls and their parents separately filled in questionnaires covering a wide range of health related topics. In addition, all participants were physically examined and interviewed by a study team led by a physician. The overall response rate was 66.6%. The present analyses are restricted to adolescents aged 11 to 17 years (n = 6 813; 51.3% male) for whom self-reported PA and pubertal status were assessed.

The study was approved by the ethics committee of the Medical University of Berlin and the Federal Office for the Protection of Data.

### Measures

#### Physical activity (PA)

Boys and girls were asked how often they are physically active in their leisure time in such a way they sweat or breathe hard (e.g. by sports or bicycling). Possible answers were: "about every day", "about 3 to 5 times a week", "about once or twice a week", "about once or twice a month", or "never". Since the last two categories were rare (5.5% and 10.0%, respectively), they were combined for the main analyses. "About every day" served as reference group in the logistic regression models.

#### Independent variables

Decimal chronological age was calculated from examination date and date of birth and was entered as continuous predictor of PA.

Absolute pubertal status was measured by self-assessment of pubic hair with the aid of drawings that represented the stages of pubic hair development [27,28]. Boys and girls in stage 1 were classified as prepubescent, stage 2 and 3 as early/mid-puberty, and stage 4 to 6 as mature/advanced puberty. As further three-stage variables mutation (voice change) was included for boys (no change, unstable voice, deep voice) and menarche (menstruation at regular intervals, irregular or not yet) for girls. Both characteristics were assessed during interviews by the study physicians as part of the health examination [29]. For each of these variables the lowest developmental stage served as reference group within the analyses.

To classify adolescents' relative pubertal timing, stage of pubic hair development (1-6) and mutation/menarche (0, 1, 2) were summed up. The resulting score was classified into tertiles (late, average and early maturation) for each one-year age group, separately for boys and girls. For pubertal timing 'average' (the middle age-specific tertile) was taken as the reference category.

To assess body dissatisfaction (BDS), adolescents were asked whether they think of themselves as "much too thin", "slightly too thin", "exactly the right weight", "slightly too fat", or "much too fat". Since notably fewer boys and girls categorised themselves as "too thin" than "too fat", the first two categories were combined. For the logistic regression models "exactly right" served as the reference category for BDS.

#### Covariates

Skinfold thickness (SFT) was determined by a Caliper (Holtain Ltd./UK) with a precision of 0.2 mm for the 0-40 mm range. SFT was measured at two sites: triceps and subscapular. Percentage body fat was estimated from SFT using the equations by Slaughter [30] and was treated as continuous covariate in the logistic regressions.

Socioeconomic status (SES) was determined using the so-called 'Winkler-index' developed for German health surveys [31]. This multi-dimensional index considers parents' education, occupational status, and net household income and is separated into three groups (low, medium and high). The higher score of mother and father was used per household.

Children, whose parents both immigrated or were of non-German citizenship and those who immigrated themselves and also had at least one parent of non-German origin, were classified as children with a migrant background. All others were classified as 'without migrant background' [32].

Eastern Germany (former GDR), including Berlin, and Western Germany (former FRG) were differentiated as being two regions, since both were separate states with different political and educational systems before 1990.

### Statistical analyses

According to the sampling procedure, all analyses were conducted employing complex sample procedures in SPSS 15.0 [33]. The sampling plan adjusted for deviations from representativity regarding age, sex, region, and nationality by weighting cases and adjusting standard errors to account for the clustering of the sample. Adjusted Chi-square statistics according to Rao and Scott [34] were computed to test for significance of categorical data (comparison of frequency distributions and multinomial logistic regression models) and adjusted Wald F-tests were computed for the comparison of means [35]. All statistics were computed separately for boys and girls.

### Exclusion of cases and missing values

The original sample consisted of 6 813 11 to 17-year-olds (51.3% male, weighted). We excluded 100 boys and 83 girls due to confirmed handicaps or pregnancies (3 girls), since for these adolescents other factors than the analysed variables might determine PA frequency.

A missing values analysis indicated that for the 6 630 remaining cases (51.3% male, weighted) the percentage of missing values fell well below 5% for each particular variable planned to be used in the regression models. However, up to 9.9% of cases had missing values on one or more of the analysed variables.

Missing values for continuous variables can be imputed by the MVA regression method in SPSS. We, therefore, used all potential variables of the final analyses as predictors of the imputed continuous values. This procedure resulted in n = 119 (3.3%, weighted) missing cases in girls and n = 167 (5.1%, weighted) missing cases for the logistic regression models in boys, respectively, as opposed to n = 254 (7.6%, weighted) in girls and n = 334 (10.2%, weighted) in boys before imputation.

Girls and boys with missing values on categorical variables, who were therefore excluded from the regression models, were compared to the analysed cases (n = 3 119 and n = 3 225). Cases with missing values were more likely to be boys (p = .003), to attend a school with lower educational attainment or special schools (p < .001), more often were of lower SES (p < .001) and were more likely to have a migrant background (p < .001). Excluded cases in tendency were also younger (M = 14.37 vs. 14.83 years), more likely to be in the early stages of pubic hair development, and early or late maturing compared to same-aged peers (p < .10). When sex, SES, migrant background, and school type were analysed simultaneously, only sex, SES and school type differed significantly between excluded and analysed cases (p < .05).

All analyses were rerun excluding cases with imputed values and confirmed the reported results with only minor variations. Therefore, only results for the sample with imputed values are reported.

### Data screening

Completed data were screened for multicollinearity and linearity in the logits [36]. There was no indication of problems with multicollinearity. Linearity in the logits was tested for the continuous predictors age and percentage body fat and there was no indication of deviance from linearity.

### Main analyses

Descriptive statistics were computed separately for girls and boys, and sex-specific distributions were compared. To examine bivariate associations between the potential predictors and PA, separate logistic regressions were computed first for each predictor, and the unadjusted ORs and 95%-confidence intervals are reported.

To estimate the possible dependency of PA on chronological age, absolute pubertal status and relative pubertal timing and the possible mediating effect of BDS, a series of logistic regression models was calculated separately for boys and girls.

Since PA frequency was significantly associated with SES, migrant background, and geographical region, at least in girls [5], and body fat was associated with both PA and pubertal maturation as well as with BDS, these variables were included as covariates in all models. Hence, a model (model 0) with demographic variables and body fat only (but excluding chronological age) was calculated first and served as baseline model for our analyses.

Then the other predictors were entered in the following sequence: chronological age (model 1), pubertal status (pubic hair stage plus menarche for girls and mutation for boys, respectively - model 2), and relative pubertal timing (model 3). BDS was entered in model 4. In the last step all possible two-way-interactions between the independent variables were first added individually and the significant interactions were then tested simultaneously. The final model (model 5) contains all independent variables plus all two-way-interactions significant at alpha = 5%.

Possible indirect effects of puberty on PA mediated by BDS were determined employing joint significance tests for the effects of puberty on BDS (alpha) and the effect of BDS on PA adjusted for puberty (beta). A significant indirect effect is thereby inferred if both tested effects are jointly significant [37].

Since Likelihood-ratio-tests are not available for complex samples procedures, the tested models were compared on the basis of Nagelkerke's pseudo R^2 ^and the significance of individual predictors. We were especially interested in changes of significant effects after adjustment for further predictors and the additional explanatory value of these added variables.

In all analyses p ≤ .05 was considered statistically significant. P-values for multiple tests were adjusted using a Šidák sequential procedure [38].

## Results

### Sample description

Descriptive statistics for the whole sample and for boys and girls separately are given in table 1. More than half of the sample indicated PA three times a week or more often, but only one quarter was regularly physically active on a daily basis. Girls were significantly more often in the groups with low PA than boys.

**Table 1:** Sample description^1^

Variable	Girls (n = 3 238)	Boys (n = 3 392)	Total (n = 6 630)
**Age: M (SE)**	14.62 (0.023)	14.62 (0.024)	14.62 (0.018)

**Percentage body fat: M (SE)**	26.26 (0.181) ***	19.39 (0.230) ***	22.74 (0.166)

**Region: n (%)**			

East (former GDR)	1099 (18.4)	1111 (18.5)	2210 (18.5)
West (former FRG)	2139 (81.6)	2281 (81.5)	4420 (81.5)

**Socioeconomic status (SES): n (%)**			

low	883 (27.8)	945 (28.2)	1828 (28.0)
medium	1568 (47.5)	1635 (46.8)	3203 (47.1)
high	787 (24.7)	812 (24.9)	1599 (24.8)

**Migrant background: n (%)**			

non-migrant	2747 (82.6)	2851 (82.3)	5598 (82.5)
migrant	488 (17.4)	540 (17.7)	1028 (17.5)

Pubic hair stage***: n (%)			

prepubescent (stage 1)	181 (4.9)	239 (6.0)	420 (5.5)
early/mid-puberty (stage 2-3)	476 (13.1)	971 (26.0)	1447 (19.7)
advanced/mature (stage 4-6)	2531 (82.0)	2108 (67.9)	4639 (74.8)

**Menarche (girls)/voice change (boys): n (%)**			

regular menses/deep voice	1685 (54.8)	1307 (44.1)	n/a
irregular menses/fluctuating voice	627 (20.6)	788 (22.9)	n/a
no menses/no change	904 (24.6)	1242 (33.1)	n/a

Relative pubertal timing**: n (%)			

early	696 (20.8)	623 (17.2)	1319 (19.0)
average	1845 (59.7)	2013 (63.5)	3858 (61.7)
late	640 (19.5)	670 (19.2)	1310 (19.3)

Body dissatisfaction (BDS) ***: n (%)			

much too thin	56 (1.7)	103 (3.1)	159 (2.4)
too thin	243 (7.2)	547 (17.1)	790 (12.3)
exactly right weight	1169 (36.4)	1519 (44.5)	2688 (40.5)
too fat	1419 (44.5)	1021 (30.6)	2440 (37.4)
much too fat	318 (10.1)	158 (4.7)	476 (7.3)

PA frequency***: n (%)			

nearly every day	582 (17.5)	987 (28.3)	1569 (23.0)
3-5 times/week	833 (26.5)	1201 (36.6)	2034 (31.7)
about 1-2 times/week	1104 (35.0)	831 (25.0)	1935 (29.9)
about 1-2 times/month	238 (7.3)	118 (3.8)	356 (5.5)
never	435 (13.9)	194 (6.3)	629 (10.0)

^1^Some values of categorical variables do not sum up to n = 6 630 because of individual missing values; n = total number unweighted, % = percent of weighted sample; M = mean; SE = standard error of mean adjusted for the sampling plan.

PA = physical activity

*** boys and girls significantly different, p ≤ .001

** boys and girls significantly different, p ≤ .01

Most of the girls and boys were in the advanced pubertal stages. With a similar age distribution girls were significantly more often in a later developmental stage than boys. The average time between stage 1 (prepubescent) and early/mid-puberty (stage 2-3) was 0.80 years in boys and 0.78 years in girls and from early/mid-puberty to advanced/mature was 2.9 years in boys and 2.6 years in girls, respectively.

About half of the girls judged themselves as "too fat" or "much too fat", in comparison to about one third of the boys.

### Univariate analyses

For girls all tested predictors showed significant univariate associations with PA (see crude ORs in table 2). The chance to be less physically active significantly rose with age, with an increase in the chance to be inactive (once or twice a month or less) of nearly 1.5 times per year of age. Both puberty variables were also significantly associated with PA, with girls in later absolute developmental stages being more likely to be inactive. Relatively late maturing girls generally were less likely to be inactive, while no significant effect was found for early maturing girls. The ORs for BDS showed a higher risk of being inactive for girls dissatisfied with their weight, especially for those judging themselves as "(much) too fat".

**Table 2:** Crude odds ratios (unadj.OR) and adjusted odds ratios (adj. OR) for the prediction of physical activity (PA) in the final logistic regression model (model 5) for girls (n = 3 119)^1^

	3-5 times/week vs. nearly every day		1-2 times/week vs. nearly every day		1-2 times/month or less vs. nearly every day	
Predictors	unadj. OR (95% CI)	adj. OR (95% CI)	unadj. OR (95% CI)	adj. OR (95% CI)	unadj. OR (95% CI)	adj. OR (95% CI)
**Age **(OR per year)	**1.09 (1.02-1.18)**	1.03 (0.91-1.17)	**1.22 (1.15-1.30)**	**1.18 (1.06-1.31)**	**1.46 (1.36-1.57)**	**1.35 (1.20-1.52)**

**Pubic hair stage**						

advanced/mature	**1.83 (1.15-2.94)**	1.50 (0.76-2.98)	**2.39 (1.45-3.93)**	1.68 (0.83-3.42)	**5.20 (2.71-9.98)**	0.96 (0.38-2.42))
early/mid-puberty	1.34 (0.82-2.17)	1.19 (0.69-2.08)	1.13 (0.66-1.93)	1.11 (0.60-2.03)	1.57 (0.77-3.23)	0.93 (0.42-2.04)
prepubescent (ref.)	1.00	1.00	1.00	1.00	1.00	1.00

**Menarche**						

regular menses	**1.35 (1.04-1.76)**	0.99 (0.63-1.55)	**1.86 (1.45-2.40)**	0.85 (0.56-1.31)	**4.94 (3.59-6.78)**	1.49 (0.89-2.49)
irregular menses	1.38 (0.95-2.01)	1.01 (0.63-1.63)	**1.95 (1.38-2.76)**	1.09 (0.71-1.67)	**3.88 (2.73-5.53)**	**1.71 (1.06-2.75)**
no menses (ref.)	1.00	1.00	1.00	1.00	1.00	1.00

**Pubertal timing**						

early	0.73 (0.53-1.00)	0.71 (0.49-1.04)	1.08 (0.83-1.40)	1.15 (0.84-1.58)	0.96 (0.71-1.28)	1.08 (0.75-1.56)
late	**0.68 (0.51-0.92)**	0.87 (0.60-1.27)	0.88 (0.67-1.15)	1.27 (0.88-1.83)	**0.55 (0.39-0.78)**	0.91 (0.55-1.50)
average (ref.)	1.00	1.00	1.00	1.00	1.00	1.00

**Body dissatisfaction (BDS)**						

much too fat	1.24 (0.78-1.96)	1.49 (0.83-2.67)	**1.51 (1.001-2.29)**	1.50 (0.88-2.55)	**2.57 (1.64-4.02)**	1.63 (0.90-2.94)
slightly too fat	**1.39 (1.03-1.86)**	**1.40 (1.01-1.94)**	**1.39 (1.07-1.79)**	1.22 (0.91-1.64)	**1.60 (1.17-2.20)**	1.18 (0.83-1.68)
(much) too thin	0.97 (0.64-1.47)	1.03 (0.67-1.58)	1.04 (0.68-1.59)	1.14 (0.73-1.79)	**1.60 (1.04-2.44)**	**2.17 (1.35-3-48)**
exactly right weight (ref.)	1.00	1.00	1.00	1.00	1.00	1.00

**^1^**Fully adjusted odds ratios are adjusted for the baseline model variables (sociodemographic variables and body fat percentage), all listed predictors, and significant two-way-interactions at alpha = 5%; significant ORs (p ≤ .05) in bold type.

In boys, PA also decreased with age. PA varied with both puberty variables: boys in advanced puberty were more likely to be less physically active, with the largest OR for the chance to be inactive after completed voice change, which was about three times higher than before voice change. In terms of relative pubertal timing, early and late maturing boys seemed to be more likely to be in the most active group. For BDS the results resembled those of girls (see crude ORs in tables 3 and 4).

**Table 3:** Crude odds ratios (unadj.OR) and adjusted odds ratios (adj. OR) for the prediction of physical activity (PA) in the final logistic regression model (model 5) for boys (n = 3 225)^1^

	3-5 times/week vs. nearly every day		1-2 times/week vs. nearly every day		1-2 times/month or less vs. nearly every day	
Predictors	unadj. OR (95% CI)	adj. OR (95% CI)	unadj. OR (95% CI)	adj. OR (95% CI)	unadj. OR (95% CI)	adj. OR (95% CI)
**Age **(OR per year)	**1.11 (1.06-1.16)**	1.14 (0.99-1.31)*	**1.15 (1.09-1.21)**	1.19 (1.05-1.34)*	**1.36 (1.24-1.48)**	1.71 (1.43-2.04)*

**Pubic hair stage**						

advanced/mature	**1.64 (1.07-2.53)**	0.81 (0.41-1.63)*	1.51 (0.97-2.36)	0.70 (0.34-1.44)*	**2.19 (1.11-4.29)**	0.27 (0.09-0.78)*
early/mid-puberty	1.21 (0.79-1.86)	0.84 (0.51-1.39)*	1.14 (0.74-1.76)	0.88 (0.50-1.53)*	0.81 (0.38-1.74)	0.36 (0.15-0.86)*
prepubescent (ref.)	1.00	1.00	1.00	1.00	1.00	1.00

**Voice change/mutation**						

deep voice	**1.50 (1.24-1.81)**	0.92 (0.55-1.54)	**1.63 (1.31-2.03)**	1.11 (0.69-1.81)	**2.89 (2.05-4.06)**	0.52 (0.27-1.00)
fluctuating voice	1.18 (0.91-1.53)	0.97 (0.67-1.40)	1.07 (0.80-1.41)	0.97 (0.67-1.42)	1.35 (0.89-2.03)	0.64 (0.39-1.07)
no change (ref.)	1.00	1.00	1.00	1.00	1.00	1.00

**Pubertal timing**						

early	0.78 (0.60-1.01)	0.92 (0.68-1.26)	**0.74 (0.56-0.98)**	0.97 (0.70-1.34)	0.96 (0.65-1.41)	**2.20 (1.36-3.56)**
late	**0.68 (0.50-0.92)**	0.67 (0.43-1.04)	0.81 (0.61-1.07)	0.83 (0.57-1.23)	**0.67 (0.46-0.97)**	**0.57 (0.33-0.98)**
average (ref.)	1.00	1.00	1.00	1.00	1.00	1.00

**Body dissatisfaction (BDS)**						

much too fat	1.35 (0.81-2.26)	1.09 (0.59-2.03)*	**1.80 (1.12-2.90)**	0.98 (0.54-1.77)*	**2.61 (1.48-4.58)**	1.37 (0.61-3.08)*
slightly too fat	1.20 (0.96-1.51)	1.05 (0.81-1.38)*	**1.57 (1.21-2.05)**	1.14 (0.82-1.58)*	1.33 (0.93-1.90)	0.93 (0.61-1.43)*
(much) too thin	1.23 (0.97-1.56)	1.22 (0.95-1.57)*	**1.50 (1.14-1.96)**	1.52 (1.16-2.00)*	1.40 (0.97-2.02)	1.24 (0.84-1.83)*
exactly right weight (ref.)	1.00	1.00	1.00	1.00	1.00	1.00

**^1^**Fully adjusted odds ratios are adjusted for the baseline model variables (sociodemographic variables and body fat percentage), all listed predictors, and significant two-way-interactions at alpha = 5%; significant ORs (p ≤ .05) in bold type.

* Predictor is part of a significant two-way-interaction term, odds ratios were therefore calculated separately for different strata of the predictor (see table 4); odds ratios given in this table are those of the main-effects-only model and therefore unadjusted for the interaction-terms.

**Table 4:** Adjusted ORs (95% CI) of significant two-way-interactions in the final logistic regression model (model 5) for boys (n = 3 225)

Adjusted ORs (95% CI) for pubic hair stage stratified by migrant background:									
	**3-5 times/week vs. nearly every day**			**1-2 times/week vs. nearly every day**			**1-2 times/month or less vs. nearly every day**		
**Migrant background ►►**	**non-migrant**		**migrant**	**non-migrant**		**migrant**	**non-migrant**		**migrant**

**Pubic hair stage**									

advanced/mature	0.90 (0.54-1.52)		0.40 (0.13-1.18)	0.97 (0.67-1.41)		**0.19 (0.07-0.49)**	0.37 (0.13-1.05)		**0.06 (0.01-0.34)**
early/mid-puberty	1.00 (0.69-1.45)		**0.27 (0.10-0.72)**	1.19 (0.97 - 1.46)		**0.24 (0.11-0.56)**	**0.39 (0.15-0.97)**		**0.18 (0.03-0.95)**
prepubescent (ref.)	1.00		1.00	1.00		1.00	1.00		1.00

**Adjusted ORs (95% CI) for body dissatisfaction (BDS) for different years of age (exemplary):**									

	**3-5 times/week vs. nearly every day**			**1-2 times/week vs. nearly every day**			**1-2 times/month or less vs. nearly every day**		
**Age ►►**	11	14	17	11	14	17	11	14	17

**Body dissatisfaction (BDS)**									

much too fat	1.06 (0.47-2.40)	1.07 (0.59-1.95)	1.09 (0.34-3.45)	0.93 (0.47-1.86)	0.93 (0.62-1.40)	0.92 (0.43-1.96)	2.00 (0.39-10.16)	1.51 (0.64-3.54)	1.14 (0.34-3.82)
slightly too fat	0.97 (0.62-1.53)	1.07 (0.77-1.47)	1.17 (0.69-1.98)	**0.52 (0.35-0.78)**	1.00 (0.86-1.18)	**1.91 (1.42-2.56)**	1.17 (0.48-2.85)	1.04 (0.65-1.68)	0.93 (0.52-1.67)
(much) too thin	1.11 (0.70-1.76)	1.20 (0.86-1.68)	1.31 (0.76-2.25)	1.24 (0.76-2.02)	**1.52 (1.16-1.99)**	**1.87 (1.39-2.51)**	1.88 (0.66-5.39)	1.46 (0.89-2.41)	1.14 (0.68-1.92)
exactly right weight (ref.)	1.00	1.00	1.00	1.00	1.00	1.00	1.00	1.00	1.00

### Model comparison

Results of the sequential logistic regressions are shown in tables 5 and 6 in terms of significance of individual predictors as well as Nagelkerke's pseudo R^2^, pseudo -2 log-likelihood, and correct classification rate of each model.

**Table 5:** Sequential comparison of multinomial logistic models for the prediction of physical activity frequency in girls (n = 3 119)

	*Statistics for individual predictors*								*Model statistics*			
**Model**		**Age**	**Pubic hair stage**	**Menarche**	**Pubertal timing**	**BDS**	**Region × body fat**	**SES × body fat**	**Correct classification**	**Pseudo -2 Log-Likelihood**	**Wald χ^2 ^(df) corr. for model**	**Nagelkerke's pseudo R^2^**

0**^#^**	Wald χ^2 ^(df) corrected								36.2%	9393.16	92.77 (12.99)	.045
	p-value*‚										< .001	

1	Wald χ^2 ^(df) corrected	112.22 (2.90)							36.7%	9222.36	178.55 (15.38)	.093
	p-value *	**< .001**									< .001	

2	Wald χ^2 ^(df) corrected	41.32 (2.80)	6.23 (5.74)	8.71 (5.60)					37.5%	9201.17	181.48 (24.41)	.099
	p-value*	**< .001**	.506	.152							< .001	

3	Wald χ^2 ^(df) corrected	40.28 (2.88)	5.98 (5.75)	6.94 (5.61)	16.70 (5.72)				38.1%	9177.39	185.18 (27.99)	.106
	p-value*	**< .001**	.588	.177	.480						< .001	

4	Wald χ^2 ^(df) corrected	39.54 (2.90)	5.65 (5.76)	7.85 (5.61)	17.12 (5.71)	21.38 (8.45)			38.2%	9146.57	189.62 (33.27)	.114
	p-value*	**< .001**	.635	.135	.414	**.014**					< .001	

5	Wald χ^2 ^(df) corrected	38.57 (2.90)	5.79 (5.75)	7.70 (5.60)	17.07 (5.70)	21.40 (8.43)	5.75 (2.92)	8.24 (5.68)	38.1%	9128.14	194.32 (38.08)	.119
	p-value*	**< .001**	.616	.147	.372	**.012**	**.048**	**.017**			< .001	

* Adjustment for multiple tests: Šidák sequential

^# ^model 0 = baseline model including body fat percentage and sociodemographic variables: region, SES, migrant background

Each row of the table shows the results of one tested model. Left-hand the test statistics for the independent variables are given while right-hand information on model fit is displayed.

The **corrected Wald chi-square test **tests if an individual independent variable (individual predictors) or all independent variables together (model statistics) significantly contribute to the prediction of the dependent variable; it is corrected for the sampling plan.

**Correct classification **rate is the proportion of participants for whom the tested model could correctly predict the category of the dependent variable (PA frequency).

**Pseudo -2 Log-Likelihood**: In logistic regression models are compared due to their -2 log-likelihood; since for complex samples no likelihood ratio test is available the values are only descriptive; better fitting models have smaller values.

**Nagelkerke's pseudo R^2 ^**is a measure of explained variation in the dependent variable that emulates R^2 ^from linear regression.

**Table 6:** Sequential comparison of multinomial logistic models for the prediction of physical activity frequency in boys (n = 3 225)

	*Statistics for individual predictors *								*Model statistics*			
**Model**		**Age**	**Pubic hair stage**	**Voice change**	**Pubertal timing**	**BDS**	**Migback × pubic hair stage**	**BDS × age**	**Correct classification**	**Pseudo -2** **Log-** **Likelihood**	**Wald χ^2 ^(df) corr. for model**	**Nagelkerke's pseudo R^2^**

0 **^#^**	Wald χ2 (df) corrected								36.5%	9431.11	39.09 (13.62)	.018
	p-value*										< .001	

1	Wald χ2 (df) corrected	68.82 (2.92)							37.6%	9319.74	104.58 (16.06)	.050
	p-value*	**< .001**									< .001	

2	Wald χ2 (df) corrected	22.77 (2.95)	4.81 (5.56)	1.55 (5.76)					37.7%	9310.25	104.20 (24.40)	.053
	p-value*	**< .001**	.836	.990							< .001	

3	Wald χ2 (df) corrected	34.45 (2.92)	7.58 (5.52)	5.31 (5.75	18.80 (5.54)				38.2%	9282.33	112.61 (27.93)	.060
	p-value*	**< .001**	*.068*	.284	**.007**						< .001	

4	Wald χ2 (df) corrected	33.06 (2.91)	7.14 (5.51)	5.42 (5.74)	18.70 (5.56)	9.90 (8.44)			38.5%	9267.46	115.69 (33.30)	.065
	p-value*	**< .001**	*.084*	.299	**.008**	**.022**					< .001	

5	Wald χ2 (df) corrected	28.01 (2.90)	12.70 (5.67)	6.20 (5.75)	19.01 (5.54)	13.86 (8.13)	20.50 (5.75)	14.73 (8.15)	38.3%	9217.06	126.10 (40.51)	.078
	p-value*	**< .001**	**.013**	.258	**.007**	**.017**	**.024**	**.008**			< .001	

* Adjustment for multiple tests: Šidák sequential

^# ^model 0 = baseline model including body fat percentage and sociodemographic variables: region, SES, migrant background migback = migrant background

Each row of the table shows the results of one tested model. Left-hand the test statistics for the independent variables are given while right-hand information on model fit is displayed.

The **corrected Wald chi-square test **tests if an individual independent variable (individual predictors) or all independent variables together (model statistics) significantly contribute to the prediction of the dependent variable; it is corrected for the sampling plan.

**Correct classification **rate is the proportion of participants for whom the tested model could correctly predict the category of the dependent variable (PA frequency).

**Pseudo -2 Log-Likelihood**: In logistic regression models are compared due to their -2 log-likelihood; since for complex samples no likelihood ratio test is available the values are only descriptive; better fitting models have smaller values.

**Nagelkerke's pseudo R^2 ^**is a measure of explained variation in the dependent variable that emulates R^2 ^from linear regression.

For girls, age showed the clearest association with PA nearly independent of other potential predictors and also clearly increased Nagelkerke's pseudo R^2^. None of the puberty-related variables showed a meaningful effect beyond age. BDS was entered last and had a significant independent effect on PA. Tests of indirect effects of BDS are described below.

As the final model shows, effects of body fat percentage varied by region and SES. A look at the adjusted ORs for inactivity dependent on body fat (not shown) illustrates that a higher percentage of body fat was associated with inactivity only in girls from Western Germany and those with high SES.

Predictors, that were significant in all boys' models independent of other variables, were age, relative pubertal timing, and BDS. Age again showed the largest increase in Nagelkerke's pseudo R^2^. As in girls' models, absolute pubertal development did not add significantly to the prediction beyond age. However, after entering pubertal timing, stage of pubic hair development failed significance only marginally. The significant interaction with migrant background in the final model, moreover, shows that puberty effects have to be considered separately for ethnic minorities. Unlike absolute pubertal status, relative pubertal timing, moreover, showed a clearly significant effect beyond chronological age for the whole sample of boys.

BDS was also associated with PA in boys, but its association varied with age.

### Final models

Tables 2 (girls) and 3 (boys) on the right-hand side of each column show the fully adjusted ORs of the final models. ORs that are marked by an asterisk are not adjusted for the interaction terms but are those for the main effects only model (model 4). Instead the ORs of the interacting variables are given for different strata of the effect modifiers in table 4.

The results show that the chance to be inactive (1-2 times/month or less) compared with daily PA in German girls and boys increased about 35-45% for each year of age (unadjusted). In boys, the magnitude of the fully adjusted effect rose to more than 70% per year, while the adjustment slightly reduced the age-effect in girls. There seems to be a negative effect of age on PA for both sexes that is in large part independent of other considered variables, including absolute and relative pubertal maturation.

The effects of absolute pubertal development diminished after accounting for age. In girls, there was one significant contrast for menarche that points to a higher risk of inactivity for girls with irregular menses. In boys, the effect of absolute pubertal status changed direction in the adjusted model: After taking age and other predictors into account, boys in the advanced puberty stages were clearly less likely to be inactive. The ORs stratified by migrant background (see table 4) showed that this effect was especially marked in ethnic minority boys.

While late pubertal timing reduced the risk of lower PA in girls in the univariate model, it was not significant after adjustment for other predictors. For boys, in contrast, the final adjusted model showed a more than doubled risk of inactivity for early maturers versus an almost halved risk for late maturers.

The risk of inactivity in both sexes increased with BDS. In girls, those who found themselves "(much) too fat", were more likely to be inactive. However, after adjusting for body fat the effect of a body image "(much) too thin" was more pronounced and showed the only significant association with inactivity. In boys, after adjusting for body fat and other predictors, the effect of a body image "too fat" diluted. The risk of lower PA was also only heightened for those who judged themselves "too thin" and only one comparison was still significant. In boys, however, these effects varied with age (see table 4): The likelihood of inactivity for those dissatisfied with their weight decreased with age, while the likelihood of being physically active less frequently (middle categories) was reduced for those feeling "slightly too fat" at the age of 11 but increased until the age of 17. This risk increased relatively steadily from 11 to 17 years for those with a body image "(much) too thin".

To test if BDS possibly mediates effects of puberty on PA, joint significance tests for absolute puberty and relative pubertal timing variables were performed (see additional file 1). In girls neither pubic hair stage nor menarche were significantly related to BDS when adjusted for covariates (see additional file 2) and were therefore discarded as mediators. Pubertal timing was significantly related to BDS: early maturing girls more often thought of themselves as "much too fat" (OR = 1.73; 95% CI = 1.16-2.58; p < .05, see additional file 3). BDS, in turn, was associated with PA (see tables 2 and 5 for results). However, the adjusted effects showed an increased risk of inactivity only for girls feeling "(much) too thin" (OR = 2.17; 95% CI = 1.35-3.48; p < .05) while none of the ORs for "much too fat" was significant. The assumption that puberty affects PA frequency via BDS could therefore not be confirmed in girls.

In boys, tests of joint significance showed significant associations of pubic hair stage and voice change with BDS (see additional file 2) as well as relations of BDS with PA after adjustment of puberty and covariates (p < .05). None of these associations was significant for relative pubertal timing.

After commencing pubertal development the likelihood of feeling "(much) too thin" significantly decreased (ORs for pubic hair stage and voice change from 0.32 to 0.55, see additional file 3), while the likelihood of feeling "slightly too fat" doubled for mature compared to prepubescent boys (OR = 2.19; 95% CI = 1.10-4.33). BDS was significantly associated with PA, in the way that boys who feel "(much) too thin" were at an increased risk of less PA (see tables 3 and 6). These results point to BDS mediating puberty effects on PA in boys.

## Discussion

The relationships between chronological age, pubertal maturation and PA were analysed for the first time in a large, representative sample of German adolescents. Overall, the results point to chronological age instead of absolute or relative pubertal maturation as dominant PA predictor. Puberty effects were only found in boys but were less marked than age effects. BDS also contributed to the prediction in both sexes. It did not mediate puberty effects in girls, while in boys the positive effect of pubertal maturation on PA seems to be partially mediated by a lower BDS.

### Chronological age versus absolute pubertal status

A consistent independent contribution to the prediction of PA was only accomplished by chronological age of both, girls and boys. Absolute pubertal status did not add to the prediction beyond chronological age concerning girls. For boys, after accounting for age and the other predictors, the effect of pubertal status changed direction: more mature boys were more likely to be physically active on a daily basis than prepubescent boys. However, this was attributable mainly to a much lower risk of inactivity in more mature migrant boys. Since the type of bodily changes does not differ with ethnic background, it has to be assumed that these account for higher PA levels rather indirectly (i.e. via culturally shaped social expectations or psychological reactions).

In girls, only irregular menses was associated with lower PA. Irregular periods are normal during the first 1-2 post-menarcheal years and, as such, are an indicator of ongoing pubertal development. However, there are also other reasons for irregularities than puberty that can be associated with pain and strong bleeding and may lead to lower PA in the affected girls [39]. An independent association of absolute pubertal development with infrequent PA in girls, therefore, could not be established.

A similar pattern of results as we observed for boys with exercise levels increasing with puberty, was found for the baseline data of the UK HABITS study [7]. No difference in this association was reported by ethnicity. Others [18,19] found a decline in PA with pubertal maturation in Canadian boys and girls without considering ethnicity. In a study by Bradley et al. [40] more mature US American girls reported less PA and more sedentary activities. There was, however, no such association found in boys. Activities differed by grade and ethnicity in both sexes.

Other cross-sectional [21,23] and longitudinal [22] studies from the UK could not find any associations of pubertal status with PA for any gender.

One explanation for the inconsistency of results may be a different methodology. Studies that analysed puberty effects differ in that they did or did not control for age, body fat and/or relative pubertal timing. Furthermore, different measures of puberty are in use, each reflecting different aspects of physical maturation (i.e. growth spurt and/or various secondary sex characteristics) [17]. Moreover, measures of PA behaviour are also diverse and cover different facets of this complex behaviour. These methods all have certain advantages and deficiencies, and the concordance of results has been shown to be modest at best [41].

Not all studies that found statistically significant associations between maturity and PA among other things adjusted the analyses for age [e.g. [18,19,40]]. Consistent with the results of these studies we found pubertal status and PA clearly associated in the univariate analyses. However, our multivariate models show that age markedly confounded this relation. There are also unadjusted analyses that did not find any associations [21,22]. Yet, these were restricted to a narrow age range.

However, in case of the HABITS study, cross-sectional analyses that used similar self-report measures found boys' PA levels decline with maturity after accounting for age effects [7], whereas we observed PA levels increasing with pubertal maturation. One explanation might be that the HABIT study measured PA by taking into account total days of PA per week (including school sports) versus exercising on weekends (yes/no) while our index of PA frequency was restricted to PA beyond physical education classes. Together, these results indicate that associations with pubertal maturation are existent only for boys' leisure time PA.

To what extent associations with pubertal status differ by ethnicity may also depend on the culture of origin as well as the culture of the study country. The situation of migrants in Germany, who set up a very heterogeneous group, may not be directly comparable to the situation of black people in the US or UK. Anyhow, our results indicate that ethnicity or culture to some extent influences the relation between puberty and PA. Attitudes towards maturation culturally differ [17,42] and may, therefore, also shape effects of puberty on PA.

### Relative pubertal timing

We found an independent effect of pubertal timing for boys. While early maturers had a more than doubled risk of being inactive than average maturing boys, this risk was nearly halved in late maturers. This result is in line with the hypothesis that early maturing adolescents are at a higher risk to behave in unhealthy ways [6,43] but discredits the "maturational deviance hypothesis" which states that both, early as well as late maturers, tend to behave in more health-risky ways. One reason for early maturers to behave more like older adolescents is, that because of a more mature appearance, they tend to be treated like adults by peers, parents, and other adults, including expectations to behave in more mature ways. More mature looking adolescents also tend to have older friends whose interests they may adopt - including sedentary leisure activities [10,43].

A study by Kemper and colleagues [44] showed higher activity levels in late maturing versus early maturing adolescents for both sexes. By contrast, other studies did not find associations between pubertal timing and PA after controlling for age [24,45].

In longitudinal analyses of the HABITS study [16], contrary to our results, early maturing boys had statistically significant higher rates of PA compared to average or late-maturing boys after controlling for age and other sociodemographic variables. Early maturing girls had significantly higher rates of PA compared to average-developing girls in early adolescence but this effect attenuated over the time. Longitudinal data from another study indicated that early timing of puberty at age 11 in girls predicted lower levels of objectively measured PA at age 13 [20].

In summary, the little evidence available for timing effects also turns out highly inconsistent. Again, this may be due to the diverse methodology of the studies, where timing and PA are measured in different ways and different covariates are controlled [46]. Furthermore, it is worth mentioning that the classification in pubertal timing groups in our and most other studies was based on within-sample comparisons. These differ between samples and are not equivalent to accelerated or retarded puberty in clinical terms.

All in all, our interpretation is that physical changes during puberty in males facilitate PA. The association of inactivity with early pubertal timing may be explained by more mature boys adopting interests of their older friends as we have pointed out before.

### Body dissatisfaction (BDS)

We found an independent association of BDS with lower PA for both sexes. While in the univariate models boys and girls who judged themselves as "much too fat" most notably were affected by a higher risk of less frequent PA, after accounting for body fat percentage this risk was far more pronounced in adolescents with a body image "(much) too thin". This result is explained by the fact that many girls and boys, who rated their bodies "too fat", indeed, had higher body fat percentages (data not shown). Nevertheless, BDS had an independent association, and it may be in part artificial to separate this effect from actual body composition. On the other hand, the lack of controlling for objective weight in studies on body image and health behaviour has been criticised [47].

Associations of BDS or a negative body image with low PA levels have been observed in a number of previous studies of adolescents. For example, Niven et al. [21] found significant associations of all subscales of the Children and Youth's Physical Self-Perception Profile with self-reported PA in early adolescent girls. However, longitudinal analyses [22] for the same sample revealed no significant association between changes in perceived body attractiveness and PA changes over 12 months. Neumark-Sztainer and colleagues [48] found from Project EAT that youths' BDS predicted lower PA levels after five years. After adjusting for BMI, this longitudinal association remained significant only in girls.

We found no indications of a mediating function of BDS that would suggest psychological reactions to physical changes as cause of inactivity in girls. Although relative pubertal timing predicted BDS, and BDS in turn predicted PA, the associations were for different forms of dissatisfaction (i.e. early maturing girls feeling too fat while girls feeling too thin were more likely to be inactive).

This result is in line with the study by Simon et al. [7], who also found no hints that effects of puberty on PA behaviour are mediated by psychological factors (stress and psychological difficulties). It is contrary to results of other studies that documented such a psychological mediation of puberty effects in girls [25,49]. In one study, perceived body attractiveness clearly mediated maturity effects on PA [49]. However, in the study by Davison et al [25] body esteem, which was similar to our measure of BDS, was not among the variables that qualified as a mediator.

Results for boys point to a partially mediated effect: puberty was associated with a decrease in feeling "too thin" and feeling too thin was associated with an increase in the odds of infrequent PA. This finding is in accordance with the presumption that pubertal development, encompassing muscular development, for boys fosters PA by lowering the risk of BDS due to feeling "too thin" (i.e. insufficiently muscular).

In fact, even if girls in general are less satisfied with their bodies, BDS seems to be highly relevant for adolescent boys, too [50]. However, while girls most often feel being too fat, boys may be dissatisfied when feeling too fat as well as too thin or falling short of the muscular male ideal, respectively [13,51,52].

Nevertheless, it is not clear if lower PA levels determine negative physical self-perceptions or if, vice versa, a negative body image leads to lower PA levels. Both direction of effects seem plausible and may be, in fact, contributing to the associations found [51]. In addition, BDS may also act as a motivator for exercising and, as such, the direction of the relationship may be opposite in subgroups.

Either way, body image seems to be an important factor during the adolescent years, which is associated with pubertal development and timing, PA, as well as with body composition.

Future research may untangle how adolescents with a negative body image (and/or with a high percentage of body fat) may be best motivated for PA and how changes in body image affect PA. Overweight as well as a negative body image may both act as PA impediments and motives at once [48,51].

### Strengths and limitations

The study is characterised by several strengths. First, data from a large representative sample were used. The KiGGS study will further be continued as a longitudinal cohort study, which implies that results from the reported survey may be verified longitudinally. Second, the employed questionnaires were extensively pretested and validated [26]. Third, the anthropometric measures were taken by trained medical professionals, and instead of BMI, which often is used as a proxy for adiposity, body fat percentage was used as a more direct measure. Last but not least, most of the existing studies, that linked aspects of body image to PA behaviour, were confined to female samples. Since our sample consisted of both sexes, it was possible to compare the results of boys and girls.

There are, however, also some limitations that have to be taken into account. The main variables studied, namely PA and pubertal maturation, were self-reported by the adolescents. It is well recognised, that the reliability of PA self-reports is limited [53]. The use of accurate objective measures (e.g. accelerometers) is preferable, but often not feasible in large epidemiological studies. Even so, a screening measure similar to the one we used performed best compared to other short measures practicable in large epidemiological surveys [54].

The possibility of assessing pubertal maturation by clinical examination of secondary sex characteristics was discarded in the KiGGS study because of a low participation rate in the pilot test, especially for girls with migrant background [29]. However, as a review by Coleman and Coleman [55] showed, self-assessment of the development of secondary sex characteristics with the aid of drawings is a relative good proxy.

We only used two aspects of pubertal status in our analyses. For a comprehensive picture of developmental status other measures like age at peak height velocity, hormone levels or ratings of further secondary sex characteristics would have to be considered and might come up with a different picture. Furthermore, it is to be considered that due to sex-specific differences in timing and order of the appearance of secondary sex characteristics, boys and girls within the same pubic hair stage need not to be of the same maturity status [56].

Only cross sectional data were analysed. It is, therefore, not possible to infer the causal direction of the found associations. However, in the case of chronological age a reverse causal influence can be ruled out.

BDS was the only psychological variable that was examined as possible mediator of puberty effects on PA. It may be that in girls other constructs would still explain associations found, as they did in other studies [7,25,49]. More studies are needed that compare sex-specific BDS effects. Since the concept of mediation implies a causal chain, the aspect of causality in relation to the role of BDS should also be clarified further.

## Conclusions

In summary, we conclude that chronological age, in general, is a far more important predictor of PA in German adolescents than absolute pubertal status or relative pubertal timing.

Our study confirms other international research which consistently showed that PA declines with age during the teenage years [5,57,58]. It cuts across the presumption that this decline is caused by physiological changes during puberty. Effects of absolute pubertal status found for boys were in the positive direction and far weaker than the effect of chronological age. Relative pubertal timing effects were shown for subgroups, but these could not explain why adolescents' PA levels on average decline with age. Body (i.e. weight) dissatisfaction seems to be an important PA predictor in its own right as well as a possible mediator of positive puberty effects on boys PA.

Since the current evidence in total argues that only a small proportion of the variance in PA can be explained by physiological processes, factors from other levels (social or psychological) must cause the PA decline. Variables, that seem to play a significant role for youths' PA behaviour but were not covered in this study, include social support or influences by peers, siblings or parents [59,60] as well as increasing time requirements for school/education, changing social expectations, and interests [11,45,61]. These factors should be analysed in conjunction with chronological age in future studies.

## List of Abbreviations used

BDS: body dissatisfaction; OR: odds ratio; PA: physical activity; SES: socioeconomic status.

## Competing interests

The authors declare that they have no competing interests.

## Authors' contributions

EF conceived of the analyses, performed the statistical analyses and drafted the manuscript. JB participated in drafting the manuscript and interpretation of the results. TL participated in the design and coordination of the KiGGS study and helped to interpret the results. PK supervised the analyses and helped to interpret the results and drafting of the manuscript. All authors read and approved the final manuscript.

## Supplementary Material

Additional file 1 **Model of the direct and mediated effects**. Figure - Model of the direct (tau) and mediated (via BDS - alpha, beta) effects of puberty on PA. Click here for file

Additional file 2 **Table 7**. Wald chi-square tests of puberty effects on body dissatisfaction (alpha). Click here for file

Additional file 3 **Table 8**. ORs for puberty effects on body dissatisfaction (alpha). Click here for file

## References

[B1] Strong WB Malina RM Blimkie CJR Daniels SR Dishman RK Gutin B Hergenroeder AC Must A Nixon PA Pivarnik JM Evidence based physical activity for school-age youth J Pediatr 2005 146 732 737 10.1016/j.jpeds.2005.01.055 15973308

[B2] Riddoch CJ Bo Andersen L Wedderkopp N Harro M Klasson-Heggebø L Sardinha LB Cooper AR Ekelund ULF Physical activity levels and patterns of 9- and 15-yr-old European children Med Sci Sports Exerc 2004 36 86 92 10.1249/01.MSS.0000106174.43932.92 14707773

[B3] Nader PR Bradley RH Houts RM McRitchie SL O'Brien M Moderate-to-vigorous physical activity from ages 9 to 15 years JAMA 2008 300 295 305 10.1001/jama.300.3.295 18632544

[B4] World Health Organization Europe Inequalities in young people's health Health Behaviour in School-aged Children international report from the 2005/2006 survey 2008 Copenhagen: World Health Organization Regional Office for Europe

[B5] Lampert T Mensink G Romahn N Woll A Körperlich-sportliche Aktivität von Kindern und Jugendlichen in Deutschland [Physical activity among children and adolescents in Germany. Results of the German Health Interview and Examination Survey for Children and Adolescents (KiGGS)] (in German) Bundesgesundheitsblatt 2007 50 634 642 10.1007/s00103-007-0224-8 17514447

[B6] Patton GC Viner R Pubertal transitions in health The Lancet 2007 369 1130 1139 10.1016/S0140-6736(07)60366-3 17398312

[B7] Simon AE Wardle J Jarvis MJ Steggles N Cartwright M Examining the relationship between pubertal stage, adolescent health behaviours and stress Psychol Med 2003 33 1369 1379 10.1017/S0033291703008390 14672245

[B8] McCabe MP Ricciardelli LA Finemore J The role of puberty, media and popularity with peers on strategies to increase weight, decrease weight and increase muscle tone among adolescent boys and girls J Psychosom Res 2002 52 145 153 10.1016/S0022-3999(01)00272-0 11897233

[B9] Michaud PA Suris JC Deppen A Gender-related psychological and behavioural correlates of pubertal timing in a national sample of Swiss adolescents Mol Cell Endocrinol 2006 254-255 172 178 16806671 10.1016/j.mce.2006.04.037

[B10] Steinberg L Morris AS Adolescent development Annu Rev Psychol 2001 52 83 110 10.1146/annurev.psych.52.1.83 11148300

[B11] Waylen A Wolke D Sex 'n' drugs 'n' rock 'n' roll: the meaning and social consequences of pubertal timing Eur J Endocrinol 2004 151 U151 159 10.1530/eje.0.151U151 15554900

[B12] McCabe MP Ricciardelli LA Body image dissatisfaction among males across the lifespan: A review of past literature J Psychosom Res 2004 56 675 685 10.1016/S0022-3999(03)00129-6 15193964

[B13] Cafri G Thompson JK Ricciardelli L McCabe M Smolak L Yesalis C Pursuit of the muscular ideal: Physical and psychological consequences and putative risk factors Clin Psychol Rev 2005 25 215 239 10.1016/j.cpr.2004.09.003 15642647

[B14] McCabe MP Ricciardelli LA Banfield S Body image, strategies to change muscles and weight, and puberty: Do they impact on positive and negative affect among adolescent boys and girls? Eat Behav 2001 2 129 149 10.1016/S1471-0153(01)00025-3 15001042

[B15] Biddle SJH Whitehead SH O'Donavon TM Nevill ME Correlates of participation in physical activity for adolescent girls: a systematic review of recent literature J Phys Act Health 2005 2 423 434

[B16] van Jaarsveld CHM Fidler JA Simon AE Wardle J Persistent impact of pubertal timing on trends in smoking, food choice, activity, and stress in adolescence Psychosom Med 2007 69 798 806 10.1097/PSY.0b013e3181576106 17942841

[B17] Alsaker FD Annotation: The impact of puberty J Child Psychol Psychiatry 1996 37 249 258 10.1111/j.1469-7610.1996.tb01403.x 8707909

[B18] Sherar LB Esliger DW Baxter-Jones AD Tremblay MS Age and gender differences in youth physical activity: does physical maturity matter? Med Sci Sports Exerc 2007 39 830 835 10.1249/mss.0b013e3180335c3c 17468582

[B19] Thompson AM Baxter-Jones AD Mirwald RL Bailey DA Comparison of physical activity in male and female children: does maturation matter? Med Sci Sports Exerc 2003 35 1684 1690 10.1249/01.MSS.0000089244.44914.1F 14523305

[B20] Baker BL Birch LL Trost SG Davison KK Advanced pubertal status at age 11 and lower physical activity in adolescent girls J Pediatr 2007 151 488 493 10.1016/j.jpeds.2007.04.017 17961691 PMC2531153

[B21] Niven AG Fawkner SG Knowles A-M Stephenson C Maturational differences in physical self-perceptions and the relationship with physical activity in early adolescent girls Pediatr Exerc Sci 2007 19 472 480 18089913 10.1123/pes.19.4.472

[B22] Knowles A-M Niven AG Fawkner SG Henretty JM A longitudinal examination of the influence of maturation on physical self-perceptions and the relationship with physical activity in early adolescent girls J Adolesc 2009 32 555 566 10.1016/j.adolescence.2008.06.001 18692232

[B23] Brodersen N Steptoe A Williamson S Wardle J Sociodemographic, developmental, environmental, and psychological correlates of physical activity and sedentary behavior at age 11 to 12 Ann Behav Med 2005 29 2 11 10.1207/s15324796abm2901_2 15677295

[B24] Wickel EE Eisenmann JC Welk GJ Maturity-related variation in moderate-to-vigorous physical activity among 9-14 year olds J Phys Act Health 2009 6 597 605 19953836 10.1123/jpah.6.5.597

[B25] Davison KK Werder JL Trost SG Baker BL Birch LL Why are early maturing girls less active? Links between pubertal development, psychological well-being, and physical activity among girls at ages 11 and 13 Soc Sci Med 2007 64 2391 2404 10.1016/j.socscimed.2007.02.033 17451855 PMC2067993

[B26] Kurth BM Kamtsiuris P Holling H Schlaud M Dolle R Ellert U Kahl H Knopf H Lange M Mensink G The challenge of comprehensively mapping children's health in a nation-wide health survey: Design of the German KiGGS-Study BMC Public Health 2008 8 196 10.1186/1471-2458-8-196 18533019 PMC2442072

[B27] Tanner JM Whitehouse RH Clinical longitudinal standards for height, weight, height velocity, weight velocity, and stages of puberty Arch Dis Child 1976 51 170 179 10.1136/adc.51.3.170 952550 PMC1545912

[B28] Malina RM Bouchard C Bar-Or O Growth, maturation, and physical activity 2004 2 Champaign, IL: Human Kinetics

[B29] Kahl H Schaffrath Rosario A Schlaud M Sexuelle Reifung von Kindern und Jugendlichen in Deutschland [Sexual maturation of children and adolescents in Germany. Results of the German Health Interview and Examination Survey for Children and Adolescents (KiGGS)](in German) Bundesgesundheitsblatt 2007 50 677 685 10.1007/s00103-007-0229-3 17514452

[B30] Slaughter MH Lohman TG Boileau RA Horswill CA Stillman RJ Van Loan MD Bemben DA Skinfold equations for estimation of body fatness in children and youth Hum Biol 1988 60 709 723 3224965

[B31] Winkler J Stolzenberg H Der Sozialschichtindex im Bundes-Gesundheitssurvey [Social Status Scaling in the German National Health Interview and Examination Survey](in German) Gesundheitswesen 1999 61 S178 S183 10726418

[B32] Schenk L Ellert U Neuhauser H Kinder und Jugendliche mit Migrationshintergrund in Deutschland [Children and adolescents in Germany with a migration background. Methodical aspects in the German Health Interview and Examination Survey for Children and Adolescents (KiGGS)] (in German) Bundesgesundheitsblatt 2007 50 590 599 10.1007/s00103-007-0220-z 17514443

[B33] SPSS Inc SPSS 2006 15.0.1 Chicago: SPSS Inc

[B34] Rao JNK Scott AJ On chi-squared tests for multiway contingency tables with cell proportions estimated from survey data Ann Stat 1984 12 46 60 10.1214/aos/1176346391

[B35] Thomas DR Rao JNK Small-sample comparisons of level and power for simple goodness-of-fit statistics under cluster sampling J Am Stat Assoc 1987 82 630 636 10.2307/2289475

[B36] Tabachnick BG Fidell LS Using multivariate statistics 2006 5 Boston, MA Pearson

[B37] MacKinnon DP Fairchild AJ Fritz MS Mediation Analysis Annu Rev Psychol 2007 58 593 614 10.1146/annurev.psych.58.110405.085542 16968208 PMC2819368

[B38] Holland BS Copenhaver MD An improved sequentially rejective Bonferroni test procedure Biometrics 1987 43 417 423 10.2307/2531823

[B39] Deligeoroglou E Tsimaris P Menstrual disturbances in puberty Best Pract Res Clin Obstet Gynaecol 2010 24 157 171 10.1016/j.bpobgyn.2009.11.001 20034856

[B40] Bradley CB McMurray RG Harrell JS Deng S Changes in common activities of 3rd through 10th graders: the CHIC Study Med Sci Sports Exerc 2000 32 2071 2078 10.1097/00005768-200012000-00017 11128854

[B41] Prince S Adamo K Hamel M Hardt J Gorber S Tremblay M A comparison of direct versus self-report measures for assessing physical activity in adults: a systematic review Int J Behav Nutr Phys Act 2008 5 56 10.1186/1479-5868-5-56 18990237 PMC2588639

[B42] Hayward C Sanborn K Puberty and the emergence of gender differences in psychopathology J Adolesc Health 2002 30 49 58 10.1016/S1054-139X(02)00336-1 11943575

[B43] Susman EJ Rogol A Lerner RM, Steinberg LD Puberty and psychological development Handbook of adolescent psychology 2004 2 Hoboken, NJ: Wiley 15 44

[B44] Kemper HC Post GB Twisk JW Rate of maturation during the teenage years: nutrient intake and physical activity between ages 12 and 22 Int J Sport Nutr 1997 7 229 240 9286746 10.1123/ijsn.7.3.229

[B45] Sherar LB Gyurcsik NC Humbert ML Dyck RF Fowler-Kerry S Baxter-Jones AD Activity and barriers in girls (8-16 yr) based on grade and maturity status Med Sci Sports Exerc 2009 41 87 95 19092703 10.1249/MSS.0b013e31818457e6

[B46] Sherar LB Cumming SP Eisenmann JC Baxter-Jones AD Malina RM Adolescent biological maturity and physical activity: biology meets behavior Pediatr Exerc Sci 2010 22 332 349 20814031 10.1123/pes.22.3.332

[B47] Crocker PRE Sabiston CM Kowalski KC McDonough MH Kowalski N Longitudinal assessment of the relationship between physical self-concept and health-related behavior and emotion in adolescent girls J Appl Sport Psychol 2006 18 185 200 10.1080/10413200600830257

[B48] Neumark-Sztainer D Paxton SJ Hannan PJ Haines J Story M Does body satisfaction matter? Five-year longitudinal associations between body satisfaction and health behaviors in adolescent females and males J Adolesc Health 2006 39 244 251 10.1016/j.jadohealth.2005.12.001 16857537

[B49] Cumming SP Standage M Loney T Gammon C Neville H Sherar LB Malina RM The mediating role of physical self-concept on relations between biological maturity status and physical activity in adolescent females J Adolesc 2010 34 465 473 20655102 10.1016/j.adolescence.2010.06.006

[B50] van den Berg P Mond J Eisenberg M Ackard D Neumark-Sztainer D The link between body dissatisfaction and self-esteem in adolescents: similarities across gender, age, weight status, race/ethnicity, and socioeconomic status J Adolesc Health 2010 47 290 296 10.1016/j.jadohealth.2010.02.004 20708569 PMC2923488

[B51] Grogan S Body Image and Health J Health Psychol 2006 11 523 530 10.1177/1359105306065013 16769732

[B52] Cohane GH Pope HG Body image in boys: A review of the literature Int J Eat Disord 2001 29 373 379 10.1002/eat.1033 11285574

[B53] Sallis JF Saelens BE Assessment of physical activity by self-report: status, limitations, and future directions Res Q Exerc Sport 2000 71 S1 14 10925819

[B54] Prochaska JJ Sallis JF Long B A physical activity screening measure for use with adolescents in primary care Arch Pediatr Adolesc Med 2001 155 554 559 11343497 10.1001/archpedi.155.5.554

[B55] Coleman L Coleman J The measurement of puberty: a review J Adolesc 2002 25 535 550 10.1006/jado.2002.0494 12234559

[B56] Sherar LB Baxter-Jones AD Mirwald RL Limitations to the use of secondary sex characteristics for gender comparisons Ann Hum Biol 2004 31 586 593 10.1080/03014460400001222 15739387

[B57] Sallis JF Age-related decline in physical activity: a synthesis of human and animal studies Med Sci Sports Exerc 2000 32 1598 1600 10994911 10.1097/00005768-200009000-00012

[B58] Gordon-Larsen P Nelson MC Popkin BM Longitudinal physical activity and sedentary behavior trends: Adolescence to adulthood Am J Prev Med 2004 27 277 283 15488356 10.1016/j.amepre.2004.07.006

[B59] Davison KK Jago R Change in parent and peer support across ages 9 to 15 yr and adolescent girls' physical activity Med Sci Sports Exerc 2009 41 1816 1825 10.1249/MSS.0b013e3181a278e2 19657287 PMC5489408

[B60] Ornelas I Perreira K Ayala G Parental influences on adolescent physical activity: a longitudinal study Int J Behav Nutr Phys Act 2007 4 3 10.1186/1479-5868-4-3 17274822 PMC1805507

[B61] Whitehead S Biddle S Adolescent girls' perceptions of physical activity: A focus group study Eur Phys Educ Rev 2008 14 243 262 10.1177/1356336X08090708

