# Supplementary material for: Age, puberty, body dissatisfaction, and physical activity decline in adolescents. Results of the German Health Interview and Examination Survey (KiGGS)
Source: Int J Behav Nutr Phys Act. 2011 Oct 27;8:119. doi: 10.1186/1479-5868-8-119 (PMC3231807; doi:10.1186/1479-5868-8-119)
Supplement: Additional file 1 — Model of the direct and mediated effects. Figure - Model of the direct (tau) and mediated (via BDS - alpha, beta) effects of puberty on PA. [file 1479-5868-8-119-S1.PDF]

Additional file 1

Figure: Model of the direct (tau) and mediated (via BDS - alpha, beta) effect of puberty on PA

a) **Direct effect**

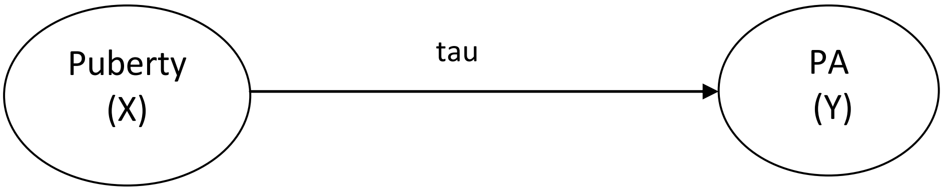

b) **Indirect effect / mediation model**

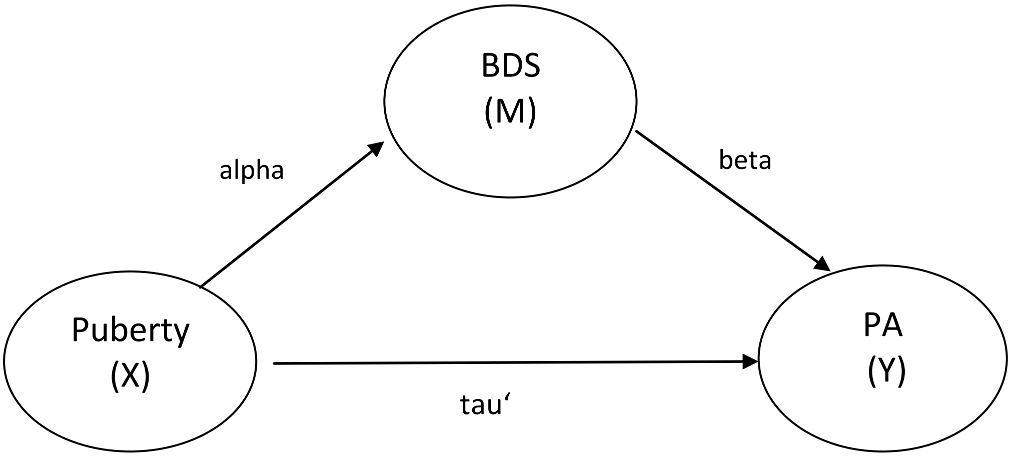

tau = direct effect of X on Y

tau’ = direct effect of X on Y adjusted for mediator M (and covariates)

alpha = effect of X on M

beta = effect of M on Y

The joint significance tests is a variant of the causal steps approach, it assumes a significant indirect effect when both alpha and beta are jointly significant.

In case of a significant mediation effect, tau’ usually is smaller than tau (partial mediation) or tau’ is near 0 (complete mediation). However, significant mediation can also occur without a significant tau effect, for instance when alpha and beta are of opposite sign or different mediators cancel out each other (suppression).
