# Supplementary material for: Age, puberty, body dissatisfaction, and physical activity decline in adolescents. Results of the German Health Interview and Examination Survey (KiGGS)
Source: Int J Behav Nutr Phys Act. 2011 Oct 27;8:119. doi: 10.1186/1479-5868-8-119 (PMC3231807; doi:10.1186/1479-5868-8-119)
Supplement: Additional file 2 — Table 7. Wald chi-square tests of puberty effects on body dissatisfaction (alpha). [file 1479-5868-8-119-S2.PDF]

Additional file 2

Table 7: Wald chi-square tests of puberty effects on body dissatisfaction (alpha)

**Girls**	*Statistics for individual predictors*		*Model statistics*			
**Predictor**	Wald χ^2 (df)	p-value*	Correct classification	Pseudo -2 Log-Likelihood	Wald χ^2 (df) corr. for model	Nagelkerke's pseudo R^2
**Pubic hair stage**						
univariate	72.76 (5.67)	<.001	47.1%	8154.656	72.76 (5.67)	0.032
adjusted	7.31 (5.61)	0.248				
**Menarche**						
univariate	87.18 (5.67)	<.001	47.9%	8122.207	87.18 (5.67)	0.042
adjusted	8.33 (5.59)	0.224				
**Pubertal timing**						
univariate	72.52 (5.66)	<.001	45.6%	8153.494	72.52 (5.66)	0.032
adjusted	9.77 (5.70)	0.040				
Adjusted model			59.7%	6373.97	538.76 (29.49)	0.458
**Boys**	*Statistics for individual predictors*		*Model statistics*			
**Predictor**	Wald χ^2 (df)	p-value*	Correct classification	Pseudo -2 Log-Likelihood	Wald χ^2 (df) corr. for model	Nagelkerke's pseudo R^2
**Pubic hair stage**						
univariate	19.67 (5.76)	0.547	44.6%	8639.11	19.67 (5.76)	0.009
adjusted	14.99 (5.61)	0.021				
**Voice change**						
univariate	23.12 (5.67)	0.001	44.6%	8630.12	23.12 (5.67)	0.011
adjusted	12.79 (5.74)	0.010				
**Pubertal timing**						
univariate	9.52 (5.57)	0.120	44.6%	8654.63	9.52 (5.57)	0.004
adjusted	10.87 (5.87)	0.331				
Adjusted model			60.0%	6425.63	537.57 (30.67)	0.506

* Adjustment for multiple tests: Šidák sequential

For the effects of body dissatisfaction on PA (beta) and puberty on PA (tau and tau') see tables 2 and 3 (Wald chi-square statistics) and tables 5 and 6 (odds ratios) in the main file.
