# Supplementary material for: Age, puberty, body dissatisfaction, and physical activity decline in adolescents. Results of the German Health Interview and Examination Survey (KiGGS)
Source: Int J Behav Nutr Phys Act. 2011 Oct 27;8:119. doi: 10.1186/1479-5868-8-119 (PMC3231807; doi:10.1186/1479-5868-8-119)
Supplement: Additional file 3 — Table 8. ORs for puberty effects on body dissatisfaction (alpha). [file 1479-5868-8-119-S3.PDF]

Additional file 3

Table 8: ORs for puberty effects on body dissatisfaction (alpha):

Girls	(Much) too thin vs. exactly right weight		Slightly too fat vs. exactly right weight		Much too fat vs. exactly right weight	
**Predictors**	unadj. OR (95% CI)	adj. OR* (95% CI)	unadj. OR (95% CI)	adj. OR* (95% CI)	unadj. OR (95% CI)	adj. OR* (95% CI)
**Pubic hair stage**						
advanced/mature	0.64 (0.39-1.05)	1.23 (0.55-2.73)	**2.41 (1.64-3.55)**	1.76 (0.94-3.29)	**4.44 (1.79-11.05)**	**4.14 (1.01-16.92)**
early/ mid-puberty	1.14 (0.64-2.04)	1.51 (0.79-2.86)	1.38 (0.89-2.19)	1.35 (0.75-2.42)	2.13 (0.74-6.08)	2.02 (0.56-8.64)
prepubescent (ref.)	1.00	1.00	1.00	1.00	1.00	1.00
**Menarche**						
regular menses	**0.51 (0.37-0.69)**	0.75 (0.42-1.33)	**1.99 (1.59-2.50)**	1.11 (0.76-1.61)	**2.16 (1.45-3.23)**	1.01 (0.56-1.81)
irregular menses	**0.61 (0.39-0.93)**	0.68 (0.38-1.22)	2.04 (0.58-2.64)	**1.43 (1.01-2.03)**	**2.11 (1.31-3.40)**	1.30 (0.67-2.52)
no menses (ref.)	1.00	1.00	1.00	1.00	1.00	1.00
**Pubertal timing**						
early	0.74 (0.46-1.17)	0.80 (0.48-1.32)	**1.26 (1.01-1.58)**	1.23 (0.93-1.64)	**1.77 (1.31-2.39)**	**1.73 (1.16-2.58)**
late	**2.03 (1.42-2.88)**	1.40 (0.85-2.31)	**0.72 (0.58-0.90)**	1.17 (0.83-1.64)	**0.62 (0.41-0.94)**	1.35 (0.71-2.60)
average (ref.)	1.00	1.00	1.00	1.00	1.00	1.00
**Boys**	**(Much) too thin vs. exactly right weight**		**Slightly too fat vs. exactly right weight**		**Much too fat vs. exactly right weight**	
**Predictors**	unadj. OR (95% CI)	adj. OR* (95% CI)	unadj. OR (95% CI)	adj. OR* (95% CI)	unadj. OR (95% CI)	adj. OR* (95% CI)
**Pubic hair stage**						
advanced/mature	1.38 (0.88-2.16)	**0.32 (0.17-0.71)**	1.33 (0.92-1.90)	**2.19 (1.10-4.33)**	0.85 (0.41-1.77)	2.35 (0.59-9.44)
early/ mid-puberty	0.82 (0.51-1.33)	**0.55 (0.31-0.97)**	1.26 (0.85-1.85)	1.62 (0.98-2.68)	0.93 (0.45-1.95)	1.31 (0.48-3.57)
prepubescent (ref.)	1.00	1.00	1.00	1.00	1.00	1.00
**Voice change/mutation**						
deep voice	**1.66 (1.27-2.17)**	**0.45 (0.27-0.74)**	1.01 (0.81-1.27)	0.87 (0.52-1.46)	0.71 (0.47-1.09)	0.92 (0.37-2.29)
fluctuating voice	1.18 (0.87-1.60)	**0.54 (0.37-0.80)**	1.08 (0.84-1.38)	1.13 (0.78-1.63)	1.02 (0.63-1.65)	1.45 (0.72-2.94)
no change (ref.)	1.00	1.00	1.00	1.00	1.00	1.00
**Pubertal timing**						
early	**0.73 (0.55-0.96)**	1.39 (0.98-1.98)	1.04 (0.85-1.27)	1.17 (0.84-1.63)	1.21 (0.72-2.09)	1.33 (0.62-2.89)
late	1.02 (0.77-1.35)	0.82 (0.56-1.18)	1.13 (0.89-1.43)	1.40 (0.96-2.04)	1.51 (0.95-2.38)	1.78 (0.91-3.50)
average (ref.)	1.00	1.00	1.00	1.00	1.00	1.00

* Adjusted ORs are the ORs that relate BDS to puberty variables adjusted for all other variables of the final model (model 5) except of physical activity (PA); significant ORs in bold type.
